# Complete Genome Sequence of the Bacterium Serratia marcescens SGAir0764, Isolated from Singapore Air

**DOI:** 10.1128/genomeA.00637-18

**Published:** 2018-07-05

**Authors:** Nicolas E. Gaultier, Ana Carolina M. Junqueira, Akira Uchida, Rikky W. Purbojati, James N. I. Houghton, Caroline Chénard, Anthony Wong, Megan E. Clare, Kavita K. Kushwaha, Deepa Panicker, Alexander Putra, Carmon Kee, Balakrishnan N. V. Premkrishnan, Cassie E. Heinle, Serene B. Y. Lim, Vineeth Kodengil Vettath, Daniela I. Drautz-Moses, Stephan C. Schuster

**Affiliations:** a Singapore Centre for Environmental Life Sciences Engineering, Nanyang Technological University, Singapore; b Departamento de Genética, Instituto de Biologia, Universidade Federal do Rio de Janeiro, Rio de Janeiro, Brazil; c Asian School of the Environment, Nanyang Technological University, Singapore

## Abstract

Serratia marcescens strain SGAir0764 was isolated from a tropical air sample collected in Singapore. The complete genome, sequenced on the PacBio RS II platform, consists of one chromosome with 5.1 Mb and one plasmid with 76.4 kb. Genome annotation predicts 4,723 protein-coding genes, 89 tRNAs, and 22 rRNAs.

## Abstract (precis)

Serratia marcescens strain SGAir0764 was isolated from a tropical air sample collected in Singapore. The complete genome, sequenced on the PacBio RS II platform, consists of one chromosome with 5.1 Mb and one plasmid with 76.4 kb.

## GENOME ANNOUNCEMENT

Serratia marcescens is a rod-shaped, facultatively anaerobic, Gram-negative bacterium (1) classified in the family Enterobacteriaceae. It was first reported in 1819 by Bizio, an Italian pharmacist, that the spontaneous bloody discoloration of polenta (cornmeal mush) was “due to the seeds of some microscopic fungus present in air” (2, 3). Since then, Serratia marcescens has been reported as an opportunistic bacterium that may cause urinary tract and other nosocomial infections (4, 5). In addition, environmental strains were isolated from a variety of ecological niches, such as soil, water, air, plants, and animals (6).

Strain SGAir0764 was isolated from an air sample collected in Singapore (global position system coordinates 1.346°N, 103.680°E). Airborne particles were impacted onto Trypticase soy agar (TSA; Becton, Dickinson, USA) using the Spin Air air sampler (IUL, Spain). After cultivation on TSA at 30°C, isolation of colonies was performed by streaking until an axenic culture was obtained. A single colony was incubated and cultured in Luria-Bertani broth at 30°C overnight prior to extraction. Genomic DNA was isolated using the Wizard genomic DNA purification kit (Promega, USA) according to the manufacturer’s protocol. Library construction was performed with the SMRTbell template prep kit 1.0 (Pacific Biosciences, USA), followed by single-molecule real-time (SMRT) sequencing on the PacBio RS II platform.

SMRT sequencing generated a total of 346,979 subreads, which were used for *de novo* assembly with Hierarchical Genome Assembly Process (HGAP) version 3 (7), which is included in the PacBio SMRT analysis 2.3.0 package. Polishing and error correction were performed with Quiver (7) and Pilon version 1.16 (8), respectively. Two contigs were generated from the consensus assembly, one chromosome with 5,142,714 bp (518-fold coverage) and a mean G+C content of 59.5% and one plasmid with 76,484 bp (186-fold coverage).

Taxonomical identification using the average nucleotide identity (ANI) method with Microbial Species Identifier (MiSI) (9) revealed a 99.0% similarity with the available reference draft genome of Serratia marcescens strain ATCC 13880 (GenBank assembly accession number GCA_000735445).

The genome was annotated using NCBI’s Prokaryotic Genome Annotation Pipeline (PGAP) version 4.2 (10). A total of 5,011 genes were predicted, including 4,723 protein-coding genes (PCGs), 22 rRNA subunits (8 genes for 5S, and 7 genes each for 16S and 23S rRNA subunits), 89 tRNAs, 12 noncoding RNAs, and 165 pseudogenes. Functional annotation with Rapid Annotations using Subsystems Technology (RAST) (11–13) showed that 112 genes were associated with virulence, disease, and defense. Of those genes, 87 were potentially linked to resistance to antibiotics and toxic compounds. This repertoire of genes may confer multidrug resistance to this bacterial strain and contribute to the incidence of nosocomial infections. Seventy-six genes were found to be related to phosphorus metabolism, thus reflecting the activity of Serratia marcescens in solubilizing this element.

### Accession number(s).

The complete genome sequence of Serratia marcescens strain SGAir0764 and its plasmid have been deposited in DDBJ/EMBL/GenBank under the accession numbers CP027300 and CP027301, respectively.
